# Cholest-5-en-3β-yl 3-(4-eth­oxy­phen­yl)prop-2-enoate

**DOI:** 10.1107/S160053681202452X

**Published:** 2012-06-13

**Authors:** Bernhard Bugenhagen, Ariane Munk, Volkmar Vill, Yosef Al-Jasem, Thies Thiemann

**Affiliations:** a Institute of Inorganic Chemistry, University of Hamburg, Hamburg, Germany; b Institute of Organic Chemistry, University of Hamburg, Hamburg, Germany; c Department of Chemical Engineering, United Arab Emirates University, Al Ain, Abu Dhabi, United Arab Emirates; d Department of Chemistry, United Arab Emirates University, Al Ain, Abu Dhabi, United Arab Emirates

## Abstract

In the asymmetric unit of the title compound, C_38_H_56_O_3_, there are two symmetry-independent mol­ecules that differ in the rotation angle along the C—O bond between the 3-(4-eth­oxy­phen­yl)prop-2-enoate and cholest-5-en-3β-yl groups by 169.3 (3)°. In both mol­ecules, steroid ring *B* adopts a half-chair conformation, rings *A* and *C* adopt a chair conformation and ring *D* exists in an envelope form. The two symmetry-independent mol­ecules pack in the crystal into separate layers parallel to (-102) with their long axis parallel to the [201] direction. Short inter­molecular C—H⋯O and C—H⋯π contacts are observed.

## Related literature

For the preparation of the title compound, see: Thiemann *et al.* (2011 ▶). For applications of this class of compounds, see: Vora (1976 ▶); Kutulya *et al.* (1983 ▶); Tanaka *et al.* (1981 ▶); Dong *et al.* (2010 ▶). For ring conformational analysis, see: Cremer & Pople (1975 ▶); Siri *et al.* (2002 ▶).
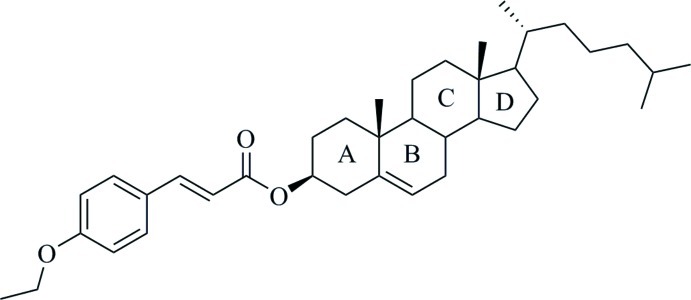


## Experimental

### 

#### Crystal data

C_38_H_56_O_3_
*M* *_r_* = 560.83
Monoclinic, 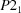

*a* = 11.6919 (5) Å
*b* = 10.5844 (4) Å
*c* = 27.3230 (11) Å
β = 101.226 (1)°
*V* = 3316.6 (2) Å^3^
*Z* = 4
Mo *K*α radiation
μ = 0.07 mm^−1^
*T* = 100 K
0.25 × 0.08 × 0.07 mm

#### Data collection

Bruker APEXII CCD area-detector diffractometer
Absorption correction: multi-scan (*SADABS*; Bruker, 2009 ▶) *T*_min_ = 0.714, *T*_max_ = 0.746
45080 measured reflections
7947 independent reflections
7272 reflections with *I* > 2σ(*I*)
*R*_int_ = 0.029

#### Refinement

*R*[*F* ^2^ > 2σ(*F* ^2^)] = 0.041
*wR*(*F* ^2^) = 0.108
*S* = 1.06
7947 reflections
751 parameters
1 restraint
H-atom parameters constrained
Δρ_max_ = 0.77 e Å^−3^
Δρ_min_ = −0.18 e Å^−3^

### 

Data collection: *APEX2* (Bruker, 2009 ▶); cell refinement: *SAINT* (Bruker, 2009 ▶); data reduction: *SAINT*; program(s) used to solve structure: *SHELXS97* (Sheldrick, 2008 ▶); program(s) used to refine structure: *SHELXL97* (Sheldrick, 2008 ▶) within *OLEX2* (Dolomanov *et al.*, 2009 ▶); molecular graphics: *PLATON* (Spek, 2009 ▶) and *Mercury* (Macrae *et al.*, 2008 ▶); software used to prepare material for publication: *SHELXL97* (Sheldrick, 2008 ▶) and *PLATON* (Spek, 2009 ▶).

## Supplementary Material

Crystal structure: contains datablock(s) global, I. DOI: 10.1107/S160053681202452X/gk2477sup1.cif

Structure factors: contains datablock(s) I. DOI: 10.1107/S160053681202452X/gk2477Isup2.hkl

Additional supplementary materials:  crystallographic information; 3D view; checkCIF report

## Figures and Tables

**Table 1:** Hydrogen-bond geometry (Å, °)

*D*—H⋯*A*	*D*—H	H⋯*A*	*D*⋯*A*	*D*—H⋯*A*
C33*B*—H33*B*⋯O2*B* ^i^	0.95	2.56	3.389 (3)	145
C37*B*—H37*C*⋯O2*B* ^i^	0.99	2.59	3.425 (3)	142
C37*A*—H37*B*⋯O2*A* ^ii^	0.99	2.40	3.362 (3)	163

Symmetry codes: (i) 
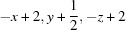
; (ii) 
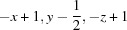
.

**Fig. 1.**
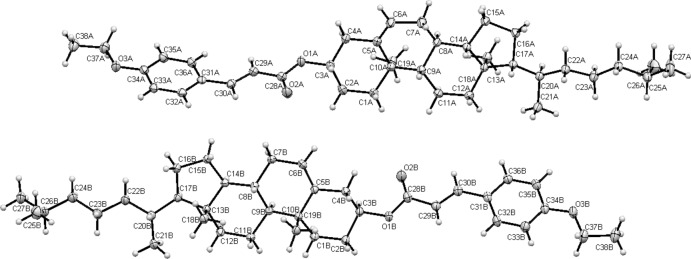
A view of molecules A, B of the title compound with the atom-numbering scheme. Displacement ellipsoids are shown at the 50% probability level.

**Fig. 2.**
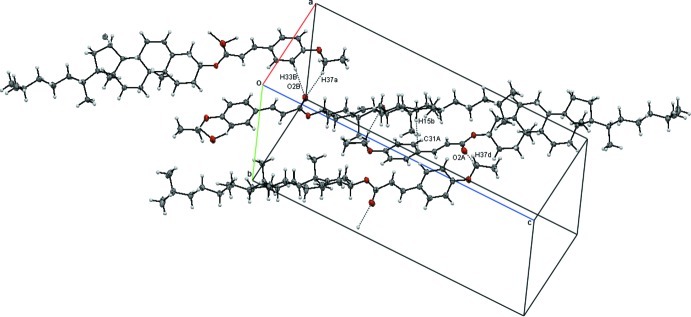
Intermolecular C—H···O contacts between molecules of the title compound. [Symmetry codes: (i) -1 + x,-1 + y,-1 + z; (ii) 1 - x,1/2 + y,1 - z; (iii) x,y,z]

**Fig. 3.**
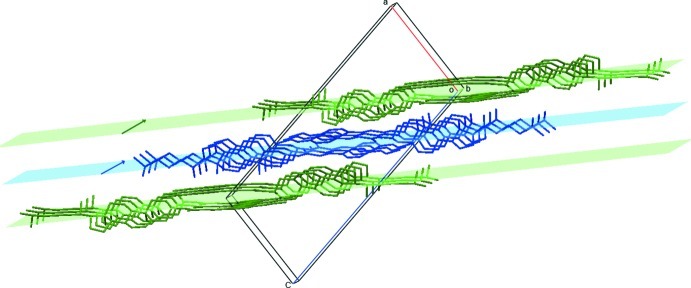
The layers formed by molecules A (blue), and B (green) are shown with the direction of the strand propagation (arrows).

## supplementary crystallographic information

### Comment

Cholesteryl cinnamates are known to exhibit chiral mesogenic phases. The interest in the influence of the substituents of the cinnamyl unit in these compounds on their phase transition behavior (Vora, 1976; Kutulya *et al.*, 1983) and on their crystal packing at room temperature and below remains unabated, also in view of the possibility of photodimerizing the substances in the crystal (Tanaka *et al.*, 1981; Dong *et al.*, 2010). For the title compound, the authors have observed the following phase transformation sequence: Cr(1) 136.2 Cr(2) 148.9 C h 270 dec., where the numbers denote temperature of the phase transition in °C. The cholesteric phase Ch undergoes decomposition at 270°C. Of special note is the Cr(1)—Cr(2) transition at 136.2°C, which proceeds through a rapid melting of Cr(1) and an immediate solidification to Cr(2). This led the authors to investigate the crystal structure Cr(1) of the compound. In the crystal, there are two symmetry independent molecules A and B that vary in their ring-conformation only slightly but differ in the rotation angle along the C—O ester bond between the 3-(4-ethoxyphenyl)-2-propenoate and cholest-5-en-3*β*-yl groups by 169.3 (3)°. In both molecules, the π system of the phenylpropenoate unit is almost planar [for A: O1A—C28A– –C31A—C36A = 3.4 (3)° and for B: O1B—C28B– –C31B—C32B = -3.3 (2)°], but the average plane of the phenylpropenoate is tilted by 55.5 (3)° *versus* the average plane defined by the carbon atoms of ring A of the cholest-5-ene framework for molecule B and by 61.1 (3)° for molecule A. In the crystal, molecules A and molecules B define separate layers parallel to (-1 0 2). Within each layer translation related molecules form columns extended along [2 0 1] with their long molecular axis collinear with this direction. Molecules in the neighbouring columns exhibit head to tail arrangement with C—H···O interactions occurring between the 4-ethoxyphenyl group and propenoate unit (Table 1). These interactions define the strands of molecules extending in the [0 1 0] direction within the layer. The molecules in neighbouring [0 1 0] strands contact *via* their steroidal fragments and the dihedral angle between the mean planes of the steroidal parts of neighboring molecules is 77.3 (3)° and 83.3 (3)° for molecules A and B, respectively. The neighboring layers are packed in such a manner that contacts are formed only between molecules A and B in a head-to-head arrangement; the dihedral angle between the steroidal mean planes of these contacting molecules is 80.3 (3)°.

A conformational analysis of rings A—D was carried out, using puckering parameters developed by Cremer and Pople (1975) (Table 2). It was found that rings A and C adopt a chair conformation, ring B adopts a half-chair conformation, and ring D adopts an envelope conformation for both molecules A and B (Siri *et al.*, 2002).

### Experimental

To a solution of triphenylphosphine (582 mg, 2.2 mmol) in CH_2_Cl_2_ (7.5 ml) is added bromotrichloromethane (900 mg, 4.5 mmol), and the resulting solution is stirred for 20 min. at rt. Thereafter, 3-(4-ethoxyphenyl)prop-2-enoic acid (4-ethoxycinnamic acid (384 mg, 2.0 mmol) is added, and the solution is heated at 50 °C for 15 min. Cholest-5-en-3*β*-ol (cholesterol, 386 mg, 1.0 mmol) is added, and after 20 min. Et_3_N (200 mg, 2.0 mmol) is added dropwise with the help of a syringe. The reaction mixture is stirred at 45 °C for 12 h. Then, it is cooled, poured into water (30 ml) and extracted with CH_2_Cl_2_ (3 *X* 15 ml). The organic phase is washed with 15w% aq. NaOH (15 ml) and subsequently with aq. HCl (1 ml conc. HCl in 7 ml of H_2_O), dried over anhydrous MgSO_4_, and evaporated *in vacuo*. Column chromatography of the residue on silica gel (eluent MtBE/hexane/CHCl_3_ 1:3:1, *R_f_* = 0.6) gives the target compound (476 mg, 85%) as a colorless solid; νmax (KBr/cm^-1^) 2946, 1713, 1631, 1602, 1511, 1469, 1313, 1252, 1163, 1009, 826; *δ*_H_ (400 MHz, CDCl_3_) 0.68 (3*H*, s, CH_3_), 0.86 (3*H*, d, ^3^*J* = 6.4 Hz, CH_3_), 0.87 (3*H*, d, ^3^*J* = 6.8 Hz, CH_3_), 0.91 (3*H*, d, ^3^*J* = 6.8 Hz, CH_3_), 1.04 (3*H*, s, CH_3_), 1.42 (3*H*, t, ^3^*J* = 7.2 Hz, CH_3_), 4.06 (2*H*, q, ^3^*J* = 7.2 Hz), 4.74 (1*H*, m), 5.40 (1*H*, m), 6.28 (1*H*, d, ^3^*J* = 16.0 Hz), 6.88 (2*H*, d, ^3^*J* = 8.8 Hz), 7.46 (2*H*, d, ^3^*J* = 8.8 Hz), 7.62 (1*H*, d, ^3^*J* = 16.0 Hz); *δ*_C_ (100.5 MHz, CDCl_3_) 11.9, 14.7, 18.7, 19.4, 21.0, 22.6, 22.8, 23.8, 24.3, 27.9, 28.0, 28.2, 31.8, 35.8, 36.2, 36.6, 37.0, 38.3, 39.5, 39.7, 42.3, 50.0, 56.1, 56.7, 63.6, 73.9, 114.7 (2 C), 116.0, 122.6, 127.1, 129.7 (2 C), 139.7, 144.2, 160.7, 166.8. The crystal was grown from hot 2-propanol.

### Refinement

All hydrogen atoms were placed in calculated positions with C—H distances of 0.95 - 1.00 Å and refined as riding with *U*_iso_(H) = *xU*_eq_(C), where *x* = 1.5 for methyl and *x* = 1.2 for all other H-atoms. The highest peak of 0.77 e Å^-3 ^is located at a distance of 1.30 Å and 1.22 Å from the alkyl group C25B and C27B atoms, respectively, however no reasonable model of disorder for the alkyl group could be found. In the absence of significant anomalous scattering effects Friedel pairs were merged as equivalent data. The absolute structure is based on the known absolute configuration of cholest-5-en-3*β*-ol used for the synthesis.

### Crystal data

**Table:**

C_38_H_56_O_3_	*D*_x_ = 1.123 Mg m^−^^3^
*M**_r_* = 560.83	Melting point: 543 K
Monoclinic, *P*2_1_	Mo *K*α radiation, λ = 0.71073 Å
*a* = 11.6919 (5) Å	Cell parameters from 9899 reflections
*b* = 10.5844 (4) Å	θ = 2.5–27.9°
*c* = 27.3230 (11) Å	µ = 0.07 mm^−^^1^
β = 101.226 (1)°	*T* = 100 K
*V* = 3316.6 (2) Å^3^	Needle, colourless
*Z* = 4	0.25 × 0.08 × 0.07 mm
*F*(000) = 1232	

### Data collection

**Table:**

Bruker APEXII CCD area-detector diffractometer	7947 independent reflections
Radiation source: micro-focus	7272 reflections with *I* > 2σ(*I*)
Multi-layer optics monochromator	*R*_int_ = 0.029
Detector resolution: 8 pixels mm^-1^	θ_max_ = 27.5°, θ_min_ = 1.8°
ω and φ scans	*h* = −15→15
Absorption correction: multi-scan (*SADABS*; Bruker, 2009)	*k* = −13→13
*T*_min_ = 0.714, *T*_max_ = 0.746	*l* = −35→35
45080 measured reflections	

### Refinement

**Table:**

Refinement on *F*^2^	Primary atom site location: structure-invariant direct methods
Least-squares matrix: full	Secondary atom site location: difference Fourier map
*R*[*F*^2^ > 2σ(*F*^2^)] = 0.041	Hydrogen site location: inferred from neighbouring sites
*wR*(*F*^2^) = 0.108	H-atom parameters constrained
*S* = 1.06	*w* = 1/[σ^2^(*F*_o_^2^) + (0.0583*P*)^2^ + 0.8113*P*] where *P* = (*F*_o_^2^ + 2*F*_c_^2^)/3
7947 reflections	(Δ/σ)_max_ = 0.001
751 parameters	Δρ_max_ = 0.77 e Å^−^^3^
1 restraint	Δρ_min_ = −0.18 e Å^−^^3^

### Special details

**Table:**

Geometry. All e.s.d.'s (except the e.s.d. in the dihedral angle between two l.s. planes) are estimated using the full covariance matrix. The cell e.s.d.'s are taken into account individually in the estimation of e.s.d.'s in distances, angles and torsion angles; correlations between e.s.d.'s in cell parameters are only used when they are defined by crystal symmetry. An approximate (isotropic) treatment of cell e.s.d.'s is used for estimating e.s.d.'s involving l.s. planes.
Refinement. Refinement of *F*^2^ against ALL reflections. The weighted *R*-factor *wR* and goodness of fit *S* are based on *F*^2^, conventional *R*-factors *R* are based on *F*, with *F* set to zero for negative *F*^2^. The threshold expression of *F*^2^ > σ(*F*^2^) is used only for calculating *R*-factors(gt) *etc*. and is not relevant to the choice of reflections for refinement. *R*-factors based on *F*^2^ are statistically about twice as large as those based on *F*, and *R*- factors based on ALL data will be even larger.

### Fractional atomic coordinates and isotropic or equivalent isotropic displacement parameters (Å^2^)

**Table:**

	*x*	*y*	*z*	*U*_iso_*/*U*_eq_	
C1B	0.50002 (19)	0.8745 (2)	0.77777 (8)	0.0237 (4)	
H1BA	0.4478	0.9488	0.7758	0.028*	
H1BB	0.4561	0.8000	0.7859	0.028*	
C2B	0.60530 (19)	0.8956 (2)	0.82008 (8)	0.0237 (4)	
H2BA	0.6468	0.9736	0.8137	0.028*	
H2BB	0.5787	0.9059	0.8521	0.028*	
C3B	0.68674 (18)	0.7837 (2)	0.82327 (8)	0.0218 (4)	
H3B	0.6463	0.7061	0.8320	0.026*	
C4B	0.72765 (18)	0.7630 (2)	0.77432 (8)	0.0226 (4)	
H4BA	0.7769	0.8352	0.7682	0.027*	
H4BB	0.7758	0.6855	0.7768	0.027*	
C5B	0.62539 (17)	0.7504 (2)	0.73096 (7)	0.0197 (4)	
C6B	0.62078 (19)	0.6562 (2)	0.69872 (8)	0.0236 (4)	
H6B	0.6810	0.5946	0.7052	0.028*	
C7B	0.52771 (19)	0.6389 (2)	0.65267 (8)	0.0241 (5)	
H7BA	0.4774	0.5668	0.6578	0.029*	
H7BB	0.5649	0.6185	0.6241	0.029*	
C8B	0.45243 (17)	0.7572 (2)	0.64045 (7)	0.0191 (4)	
H8B	0.4980	0.8223	0.6259	0.023*	
C9B	0.42165 (17)	0.8100 (2)	0.68878 (7)	0.0188 (4)	
H9B	0.3882	0.7380	0.7051	0.023*	
C10B	0.53253 (18)	0.8536 (2)	0.72619 (7)	0.0186 (4)	
C11B	0.32670 (19)	0.9126 (2)	0.67917 (8)	0.0260 (5)	
H11C	0.2996	0.9292	0.7107	0.031*	
H11D	0.3618	0.9917	0.6695	0.031*	
C12B	0.22060 (19)	0.8796 (2)	0.63854 (8)	0.0239 (5)	
H12C	0.1773	0.8088	0.6501	0.029*	
H12D	0.1677	0.9534	0.6324	0.029*	
C13B	0.25827 (17)	0.8421 (2)	0.58983 (7)	0.0180 (4)	
C14B	0.34130 (17)	0.72835 (19)	0.60296 (7)	0.0185 (4)	
H14B	0.2978	0.6639	0.6189	0.022*	
C15B	0.35437 (18)	0.6737 (2)	0.55244 (8)	0.0213 (4)	
H15C	0.3677	0.5813	0.5548	0.026*	
H15D	0.4201	0.7140	0.5404	0.026*	
C16B	0.23677 (18)	0.7043 (2)	0.51733 (8)	0.0224 (4)	
H16C	0.1955	0.6254	0.5051	0.027*	
H16D	0.2503	0.7537	0.4882	0.027*	
C17B	0.16351 (17)	0.7823 (2)	0.54817 (7)	0.0187 (4)	
H17B	0.1182	0.7210	0.5647	0.022*	
C18B	0.31660 (19)	0.9530 (2)	0.56817 (8)	0.0233 (4)	
H18D	0.2615	1.0235	0.5612	0.035*	
H18E	0.3399	0.9265	0.5372	0.035*	
H18F	0.3857	0.9800	0.5923	0.035*	
C19B	0.5826 (2)	0.9771 (2)	0.70902 (8)	0.0249 (5)	
H19D	0.5298	1.0473	0.7122	0.037*	
H19E	0.5904	0.9689	0.6741	0.037*	
H19F	0.6593	0.9937	0.7299	0.037*	
C20B	0.07489 (18)	0.8707 (2)	0.51588 (8)	0.0221 (4)	
H20B	0.1189	0.9300	0.4979	0.026*	
C21B	0.0071 (2)	0.9492 (2)	0.54760 (9)	0.0295 (5)	
H21D	0.0585	1.0139	0.5658	0.044*	
H21E	−0.0214	0.8941	0.5714	0.044*	
H21F	−0.0592	0.9901	0.5259	0.044*	
C22B	−0.00947 (19)	0.7938 (2)	0.47650 (8)	0.0257 (5)	
H22C	0.0368	0.7371	0.4590	0.031*	
H22D	−0.0584	0.7400	0.4938	0.031*	
C23B	−0.0894 (2)	0.8743 (2)	0.43762 (9)	0.0320 (5)	
H23C	−0.1372	0.9299	0.4548	0.038*	
H23D	−0.0410	0.9289	0.4203	0.038*	
C24B	−0.1698 (2)	0.7943 (2)	0.39908 (8)	0.0272 (5)	
H24C	−0.2122	0.7340	0.4168	0.033*	
H24D	−0.1216	0.7443	0.3801	0.033*	
C25B	−0.2593 (2)	0.8704 (2)	0.36200 (8)	0.0270 (5)	
H25B	−0.2999	0.9291	0.3817	0.032*	
C26B	−0.2019 (3)	0.9504 (3)	0.32722 (10)	0.0397 (6)	
H26D	−0.1606	0.8952	0.3077	0.060*	
H26E	−0.1465	1.0090	0.3470	0.060*	
H26F	−0.2618	0.9983	0.3046	0.060*	
C27B	−0.3518 (2)	0.7833 (3)	0.33197 (9)	0.0358 (6)	
H27D	−0.3899	0.7349	0.3549	0.054*	
H27E	−0.3145	0.7250	0.3120	0.054*	
H27F	−0.4101	0.8341	0.3098	0.054*	
C28B	0.84713 (19)	0.7127 (2)	0.88528 (8)	0.0247 (5)	
C29B	0.93912 (18)	0.7553 (2)	0.92685 (8)	0.0238 (4)	
H29B	0.9521	0.8430	0.9328	0.029*	
C30B	1.00404 (18)	0.6710 (2)	0.95611 (8)	0.0243 (5)	
H30B	0.9909	0.5848	0.9471	0.029*	
C31B	1.09345 (18)	0.6961 (2)	1.00060 (8)	0.0228 (4)	
C32B	1.12289 (19)	0.8178 (2)	1.01864 (8)	0.0251 (5)	
H32B	1.0854	0.8885	1.0009	0.030*	
C33B	1.2056 (2)	0.8380 (2)	1.06179 (9)	0.0269 (5)	
H33B	1.2248	0.9215	1.0733	0.032*	
C34B	1.26024 (18)	0.7344 (2)	1.08804 (8)	0.0238 (4)	
C35B	1.23214 (19)	0.6126 (2)	1.07093 (8)	0.0257 (5)	
H35B	1.2692	0.5421	1.0889	0.031*	
C36B	1.15012 (19)	0.5942 (2)	1.02768 (8)	0.0250 (5)	
H36B	1.1319	0.5106	1.0161	0.030*	
C37B	1.3761 (2)	0.8662 (3)	1.15029 (9)	0.0325 (5)	
H37C	1.3078	0.9131	1.1573	0.039*	
H37D	1.4104	0.9148	1.1257	0.039*	
C38B	1.4655 (2)	0.8477 (3)	1.19796 (9)	0.0385 (6)	
H38D	1.4328	0.7932	1.2208	0.058*	
H38E	1.4860	0.9298	1.2138	0.058*	
H38F	1.5355	0.8080	1.1901	0.058*	
O1B	0.78630 (13)	0.81197 (15)	0.86300 (5)	0.0237 (3)	
O2B	0.82610 (16)	0.60381 (17)	0.87301 (7)	0.0356 (4)	
O3B	1.34174 (14)	0.74309 (17)	1.13115 (6)	0.0294 (4)	
C1A	0.8932 (2)	0.3266 (2)	0.77104 (9)	0.0315 (5)	
H1AA	0.8663	0.3602	0.8007	0.038*	
H1AB	0.9451	0.3906	0.7604	0.038*	
C2A	0.7868 (2)	0.3066 (3)	0.72871 (9)	0.0335 (6)	
H2AA	0.7323	0.2461	0.7396	0.040*	
H2AB	0.7453	0.3878	0.7207	0.040*	
C3A	0.82570 (19)	0.2560 (2)	0.68269 (8)	0.0267 (5)	
H3A	0.8760	0.3198	0.6700	0.032*	
C4A	0.89175 (19)	0.1329 (2)	0.69391 (8)	0.0248 (5)	
H4AA	0.9227	0.1065	0.6642	0.030*	
H4AB	0.8376	0.0665	0.7009	0.030*	
C5A	0.99232 (19)	0.1453 (2)	0.73842 (8)	0.0231 (4)	
C6A	1.0972 (2)	0.1040 (3)	0.73531 (9)	0.0332 (6)	
H6A	1.1081	0.0719	0.7041	0.040*	
C7A	1.2005 (2)	0.1042 (3)	0.77772 (9)	0.0383 (6)	
H7AA	1.2561	0.1702	0.7716	0.046*	
H7AB	1.2406	0.0216	0.7788	0.046*	
C8A	1.16655 (19)	0.1286 (2)	0.82794 (8)	0.0236 (5)	
H8A	1.1295	0.0505	0.8384	0.028*	
C9A	1.07796 (18)	0.2372 (2)	0.82321 (8)	0.0223 (4)	
H9A	1.1138	0.3102	0.8085	0.027*	
C10A	0.96339 (18)	0.2038 (2)	0.78571 (8)	0.0218 (4)	
C11A	1.05688 (19)	0.2817 (2)	0.87455 (8)	0.0259 (5)	
H11A	1.0092	0.2175	0.8877	0.031*	
H11B	1.0113	0.3611	0.8699	0.031*	
C12A	1.16956 (19)	0.3045 (2)	0.91377 (8)	0.0242 (5)	
H12A	1.2118	0.3778	0.9035	0.029*	
H12B	1.1487	0.3249	0.9463	0.029*	
C13A	1.24945 (18)	0.1887 (2)	0.91977 (8)	0.0204 (4)	
C14A	1.27365 (18)	0.1597 (2)	0.86724 (8)	0.0221 (4)	
H14A	1.3069	0.2389	0.8558	0.027*	
C15A	1.3730 (2)	0.0634 (3)	0.87590 (9)	0.0320 (6)	
H15A	1.4205	0.0701	0.8497	0.038*	
H15B	1.3424	−0.0237	0.8761	0.038*	
C16A	1.44533 (19)	0.0991 (2)	0.92745 (8)	0.0270 (5)	
H16A	1.5237	0.1290	0.9241	0.032*	
H16B	1.4546	0.0249	0.9500	0.032*	
C17A	1.37803 (18)	0.2058 (2)	0.94863 (7)	0.0212 (4)	
H17A	1.4069	0.2880	0.9377	0.025*	
C18A	1.1925 (2)	0.0769 (2)	0.94191 (9)	0.0270 (5)	
H18A	1.2458	0.0045	0.9462	0.040*	
H18B	1.1197	0.0538	0.9193	0.040*	
H18C	1.1756	0.1012	0.9744	0.040*	
C19A	0.8886 (2)	0.1098 (3)	0.80890 (9)	0.0310 (5)	
H19A	0.8561	0.1524	0.8350	0.047*	
H19B	0.9372	0.0387	0.8235	0.047*	
H19C	0.8249	0.0785	0.7830	0.047*	
C20A	1.40391 (18)	0.2050 (2)	1.00620 (8)	0.0240 (4)	
H20A	1.3777	0.1219	1.0176	0.029*	
C21A	1.3378 (2)	0.3099 (3)	1.02773 (9)	0.0325 (5)	
H21A	1.3535	0.3913	1.0132	0.049*	
H21B	1.3638	0.3132	1.0640	0.049*	
H21C	1.2540	0.2924	1.0197	0.049*	
C22A	1.53664 (19)	0.2170 (2)	1.02611 (8)	0.0259 (5)	
H22A	1.5771	0.1524	1.0095	0.031*	
H22B	1.5625	0.3009	1.0166	0.031*	
C23A	1.57473 (19)	0.2015 (3)	1.08280 (8)	0.0276 (5)	
H23A	1.5436	0.2729	1.0996	0.033*	
H23B	1.5409	0.1225	1.0932	0.033*	
C24A	1.70691 (19)	0.1971 (3)	1.09949 (8)	0.0275 (5)	
H24A	1.7411	0.2689	1.0841	0.033*	
H24B	1.7361	0.1183	1.0867	0.033*	
C25A	1.7501 (2)	0.2024 (3)	1.15630 (8)	0.0314 (5)	
H25A	1.7177	0.2808	1.1689	0.038*	
C26A	1.7076 (3)	0.0899 (3)	1.18265 (10)	0.0461 (7)	
H26A	1.7367	0.0115	1.1704	0.069*	
H26B	1.6222	0.0887	1.1758	0.069*	
H26C	1.7367	0.0970	1.2187	0.069*	
C27A	1.8831 (2)	0.2121 (4)	1.16863 (10)	0.0549 (9)	
H27A	1.9082	0.2856	1.1516	0.082*	
H27B	1.9172	0.1353	1.1574	0.082*	
H27C	1.9093	0.2215	1.2048	0.082*	
C28A	0.67884 (19)	0.3256 (2)	0.61451 (8)	0.0240 (5)	
C29A	0.57405 (18)	0.2878 (2)	0.57844 (8)	0.0236 (4)	
H29A	0.5433	0.2050	0.5795	0.028*	
C30A	0.52194 (19)	0.3705 (2)	0.54399 (8)	0.0217 (4)	
H30A	0.5531	0.4537	0.5468	0.026*	
C31A	0.42368 (18)	0.3498 (2)	0.50272 (8)	0.0204 (4)	
C32A	0.39284 (18)	0.4477 (2)	0.46799 (8)	0.0220 (4)	
H32A	0.4311	0.5270	0.4739	0.026*	
C33A	0.30829 (19)	0.4316 (2)	0.42559 (8)	0.0234 (4)	
H33A	0.2886	0.4995	0.4027	0.028*	
C34A	0.25167 (19)	0.3153 (2)	0.41630 (8)	0.0236 (4)	
C35A	0.27867 (18)	0.2172 (2)	0.45101 (8)	0.0244 (4)	
H35A	0.2390	0.1386	0.4455	0.029*	
C36A	0.36379 (18)	0.2355 (2)	0.49353 (8)	0.0235 (4)	
H36A	0.3817	0.1686	0.5169	0.028*	
C37A	0.1117 (2)	0.1912 (2)	0.36052 (9)	0.0306 (5)	
H37A	0.0605	0.1724	0.3845	0.037*	
H37B	0.1681	0.1211	0.3614	0.037*	
C38A	0.0401 (2)	0.2070 (3)	0.30852 (9)	0.0357 (6)	
H38A	−0.0136	0.2783	0.3081	0.054*	
H38B	−0.0046	0.1297	0.2987	0.054*	
H38C	0.0921	0.2233	0.2851	0.054*	
O1A	0.72293 (13)	0.22869 (15)	0.64413 (6)	0.0264 (3)	
O2A	0.72243 (15)	0.43022 (16)	0.61707 (7)	0.0346 (4)	
O3A	0.17188 (14)	0.30866 (16)	0.37293 (6)	0.0284 (4)	

### Atomic displacement parameters (Å^2^)

**Table:**

	*U*^11^	*U*^22^	*U*^33^	*U*^12^	*U*^13^	*U*^23^
C1B	0.0215 (10)	0.0300 (11)	0.0188 (10)	0.0033 (9)	0.0020 (8)	−0.0017 (9)
C2B	0.0226 (10)	0.0304 (12)	0.0168 (10)	0.0035 (9)	0.0009 (8)	−0.0018 (8)
C3B	0.0201 (10)	0.0252 (11)	0.0180 (10)	0.0001 (9)	−0.0016 (8)	0.0005 (8)
C4B	0.0184 (10)	0.0259 (11)	0.0220 (10)	0.0033 (8)	0.0006 (8)	−0.0009 (8)
C5B	0.0160 (9)	0.0242 (10)	0.0182 (10)	0.0013 (8)	0.0016 (7)	0.0006 (8)
C6B	0.0202 (10)	0.0228 (10)	0.0263 (11)	0.0058 (9)	0.0005 (8)	−0.0019 (9)
C7B	0.0228 (11)	0.0219 (11)	0.0253 (11)	0.0049 (9)	−0.0010 (9)	−0.0073 (9)
C8B	0.0181 (9)	0.0205 (10)	0.0181 (10)	0.0023 (8)	0.0021 (7)	−0.0027 (8)
C9B	0.0171 (9)	0.0219 (10)	0.0165 (9)	0.0021 (8)	0.0012 (7)	−0.0017 (8)
C10B	0.0186 (9)	0.0200 (10)	0.0165 (9)	0.0016 (8)	0.0016 (8)	−0.0016 (8)
C11B	0.0250 (11)	0.0288 (11)	0.0224 (11)	0.0098 (9)	0.0000 (9)	−0.0086 (9)
C12B	0.0201 (10)	0.0323 (12)	0.0184 (10)	0.0076 (9)	0.0019 (8)	−0.0031 (9)
C13B	0.0162 (9)	0.0197 (10)	0.0172 (9)	−0.0011 (8)	0.0011 (7)	−0.0017 (7)
C14B	0.0180 (9)	0.0183 (10)	0.0193 (10)	0.0006 (8)	0.0035 (8)	−0.0018 (8)
C15B	0.0207 (10)	0.0221 (10)	0.0201 (10)	0.0015 (8)	0.0019 (8)	−0.0052 (8)
C16B	0.0209 (10)	0.0236 (10)	0.0209 (10)	0.0002 (9)	−0.0002 (8)	−0.0034 (8)
C17B	0.0176 (9)	0.0182 (9)	0.0190 (10)	−0.0012 (8)	0.0006 (8)	−0.0006 (8)
C18B	0.0232 (10)	0.0202 (10)	0.0251 (11)	−0.0021 (9)	0.0017 (8)	0.0005 (8)
C19B	0.0266 (11)	0.0221 (11)	0.0237 (11)	−0.0021 (9)	−0.0008 (9)	0.0001 (8)
C20B	0.0209 (10)	0.0205 (10)	0.0223 (10)	−0.0002 (8)	−0.0019 (8)	0.0010 (8)
C21B	0.0250 (11)	0.0273 (11)	0.0310 (12)	0.0074 (9)	−0.0073 (9)	−0.0038 (9)
C22B	0.0239 (11)	0.0237 (11)	0.0255 (11)	−0.0004 (9)	−0.0046 (9)	−0.0013 (9)
C23B	0.0345 (13)	0.0218 (11)	0.0331 (13)	−0.0006 (10)	−0.0099 (10)	0.0002 (9)
C24B	0.0298 (11)	0.0222 (11)	0.0262 (11)	0.0009 (9)	−0.0025 (9)	0.0003 (9)
C25B	0.0320 (12)	0.0219 (11)	0.0232 (11)	0.0030 (9)	−0.0038 (9)	−0.0005 (9)
C26B	0.0471 (15)	0.0384 (14)	0.0317 (14)	−0.0009 (13)	0.0027 (11)	0.0082 (11)
C27B	0.0363 (13)	0.0354 (14)	0.0298 (13)	0.0015 (11)	−0.0078 (10)	−0.0026 (10)
C28B	0.0241 (11)	0.0260 (11)	0.0225 (10)	0.0016 (9)	0.0014 (8)	0.0044 (9)
C29B	0.0233 (10)	0.0265 (11)	0.0208 (10)	0.0004 (9)	0.0023 (8)	0.0002 (9)
C30B	0.0230 (10)	0.0266 (11)	0.0226 (10)	0.0002 (9)	0.0025 (8)	0.0011 (8)
C31B	0.0192 (10)	0.0288 (11)	0.0201 (10)	0.0013 (9)	0.0029 (8)	0.0034 (9)
C32B	0.0238 (11)	0.0267 (11)	0.0228 (11)	0.0025 (9)	0.0000 (9)	0.0065 (9)
C33B	0.0268 (11)	0.0270 (12)	0.0251 (11)	−0.0028 (9)	0.0005 (9)	0.0005 (9)
C34B	0.0178 (10)	0.0331 (12)	0.0197 (10)	0.0015 (9)	0.0017 (8)	0.0022 (9)
C35B	0.0223 (11)	0.0286 (12)	0.0248 (11)	0.0049 (9)	0.0012 (9)	0.0066 (9)
C36B	0.0239 (11)	0.0246 (11)	0.0252 (11)	0.0011 (9)	0.0015 (9)	0.0007 (9)
C37B	0.0292 (12)	0.0381 (14)	0.0271 (12)	−0.0056 (11)	−0.0024 (10)	−0.0002 (10)
C38B	0.0304 (13)	0.0540 (17)	0.0274 (13)	−0.0065 (12)	−0.0032 (10)	−0.0001 (11)
O1B	0.0229 (8)	0.0261 (8)	0.0189 (7)	0.0029 (6)	−0.0032 (6)	0.0008 (6)
O2B	0.0386 (10)	0.0265 (9)	0.0349 (9)	−0.0016 (8)	−0.0098 (8)	0.0022 (7)
O3B	0.0257 (8)	0.0342 (9)	0.0245 (8)	−0.0002 (7)	−0.0044 (6)	0.0021 (7)
C1A	0.0306 (12)	0.0264 (12)	0.0319 (12)	0.0098 (10)	−0.0073 (10)	−0.0082 (10)
C2A	0.0280 (12)	0.0333 (13)	0.0334 (13)	0.0120 (11)	−0.0079 (10)	−0.0080 (11)
C3A	0.0245 (11)	0.0241 (11)	0.0273 (11)	−0.0003 (9)	−0.0051 (9)	−0.0009 (9)
C4A	0.0241 (11)	0.0284 (11)	0.0203 (10)	0.0021 (9)	0.0002 (8)	−0.0037 (9)
C5A	0.0238 (11)	0.0239 (11)	0.0199 (10)	0.0024 (9)	0.0002 (8)	−0.0023 (8)
C6A	0.0293 (12)	0.0503 (16)	0.0187 (11)	0.0097 (11)	0.0014 (9)	−0.0082 (10)
C7A	0.0264 (12)	0.0654 (19)	0.0219 (11)	0.0152 (12)	0.0014 (9)	−0.0110 (12)
C8A	0.0207 (10)	0.0303 (12)	0.0184 (10)	0.0055 (9)	0.0007 (8)	−0.0056 (9)
C9A	0.0199 (10)	0.0240 (11)	0.0221 (10)	0.0005 (9)	0.0018 (8)	−0.0011 (8)
C10A	0.0194 (10)	0.0246 (10)	0.0207 (10)	0.0020 (9)	0.0020 (8)	−0.0035 (8)
C11A	0.0214 (10)	0.0291 (12)	0.0257 (11)	0.0069 (9)	0.0013 (8)	−0.0060 (9)
C12A	0.0233 (11)	0.0253 (11)	0.0227 (11)	0.0040 (9)	0.0011 (8)	−0.0052 (9)
C13A	0.0184 (9)	0.0233 (11)	0.0185 (10)	0.0007 (8)	0.0012 (8)	−0.0024 (8)
C14A	0.0179 (10)	0.0292 (11)	0.0187 (10)	0.0042 (9)	0.0025 (8)	−0.0040 (8)
C15A	0.0253 (11)	0.0442 (15)	0.0238 (11)	0.0124 (11)	−0.0015 (9)	−0.0097 (10)
C16A	0.0207 (10)	0.0363 (13)	0.0225 (11)	0.0087 (10)	0.0005 (8)	−0.0051 (9)
C17A	0.0192 (10)	0.0245 (10)	0.0192 (10)	0.0014 (8)	0.0020 (8)	−0.0019 (8)
C18A	0.0251 (11)	0.0289 (12)	0.0254 (11)	−0.0037 (9)	0.0012 (9)	0.0007 (9)
C19A	0.0254 (11)	0.0419 (14)	0.0248 (11)	−0.0071 (11)	0.0028 (9)	−0.0026 (10)
C20A	0.0203 (10)	0.0311 (11)	0.0200 (10)	−0.0003 (9)	0.0020 (8)	−0.0033 (9)
C21A	0.0244 (11)	0.0465 (15)	0.0245 (11)	0.0022 (11)	−0.0004 (9)	−0.0125 (11)
C22A	0.0224 (10)	0.0327 (12)	0.0211 (10)	0.0003 (10)	0.0009 (8)	−0.0028 (9)
C23A	0.0257 (11)	0.0362 (13)	0.0198 (10)	−0.0017 (10)	0.0020 (9)	−0.0016 (9)
C24A	0.0231 (11)	0.0361 (13)	0.0220 (11)	0.0039 (10)	0.0009 (9)	0.0014 (9)
C25A	0.0281 (12)	0.0430 (14)	0.0215 (11)	0.0069 (11)	0.0007 (9)	0.0014 (10)
C26A	0.0637 (19)	0.0475 (17)	0.0275 (13)	0.0134 (15)	0.0096 (13)	0.0123 (12)
C27A	0.0307 (14)	0.104 (3)	0.0260 (13)	0.0090 (17)	−0.0044 (11)	0.0008 (16)
C28A	0.0243 (11)	0.0231 (11)	0.0238 (11)	0.0044 (9)	0.0027 (9)	−0.0010 (8)
C29A	0.0229 (10)	0.0227 (10)	0.0241 (11)	0.0029 (9)	0.0019 (8)	−0.0021 (8)
C30A	0.0225 (10)	0.0227 (10)	0.0207 (10)	−0.0003 (9)	0.0060 (8)	−0.0031 (8)
C31A	0.0185 (10)	0.0235 (10)	0.0200 (10)	0.0031 (8)	0.0056 (8)	−0.0015 (8)
C32A	0.0232 (10)	0.0211 (10)	0.0225 (10)	0.0023 (9)	0.0064 (8)	−0.0008 (8)
C33A	0.0266 (11)	0.0239 (11)	0.0197 (10)	0.0049 (9)	0.0049 (8)	0.0012 (8)
C34A	0.0205 (10)	0.0276 (11)	0.0219 (10)	0.0045 (9)	0.0020 (8)	−0.0009 (9)
C35A	0.0204 (10)	0.0224 (11)	0.0290 (11)	−0.0012 (9)	0.0017 (9)	0.0001 (9)
C36A	0.0243 (11)	0.0217 (11)	0.0239 (11)	0.0022 (9)	0.0034 (8)	0.0032 (8)
C37A	0.0269 (12)	0.0319 (13)	0.0304 (12)	−0.0025 (10)	−0.0003 (9)	−0.0026 (10)
C38A	0.0282 (12)	0.0504 (16)	0.0269 (12)	−0.0013 (12)	0.0014 (10)	−0.0046 (11)
O1A	0.0242 (8)	0.0254 (8)	0.0249 (8)	0.0000 (7)	−0.0065 (6)	0.0001 (6)
O2A	0.0331 (9)	0.0245 (9)	0.0398 (10)	−0.0007 (7)	−0.0084 (7)	0.0010 (7)
O3A	0.0290 (8)	0.0287 (8)	0.0238 (8)	0.0016 (7)	−0.0042 (6)	−0.0006 (7)

### Geometric parameters (Å, º)

**Table:**

C1B—H1BA	0.9900	C1A—H1AA	0.9900
C1B—H1BB	0.9900	C1A—H1AB	0.9900
C1B—C2B	1.532 (3)	C1A—C2A	1.539 (3)
C1B—C10B	1.545 (3)	C1A—C10A	1.548 (3)
C2B—H2BA	0.9900	C2A—H2AA	0.9900
C2B—H2BB	0.9900	C2A—H2AB	0.9900
C2B—C3B	1.512 (3)	C2A—C3A	1.516 (3)
C3B—H3B	1.0000	C3A—H3A	1.0000
C3B—C4B	1.521 (3)	C3A—C4A	1.515 (3)
C3B—O1B	1.460 (2)	C3A—O1A	1.464 (2)
C4B—H4BA	0.9900	C4A—H4AA	0.9900
C4B—H4BB	0.9900	C4A—H4AB	0.9900
C4B—C5B	1.517 (3)	C4A—C5A	1.524 (3)
C5B—C6B	1.325 (3)	C5A—C6A	1.320 (3)
C5B—C10B	1.528 (3)	C5A—C10A	1.529 (3)
C6B—H6B	0.9500	C6A—H6A	0.9500
C6B—C7B	1.506 (3)	C6A—C7A	1.502 (3)
C7B—H7BA	0.9900	C7A—H7AA	0.9900
C7B—H7BB	0.9900	C7A—H7AB	0.9900
C7B—C8B	1.530 (3)	C7A—C8A	1.523 (3)
C8B—H8B	1.0000	C8A—H8A	1.0000
C8B—C9B	1.540 (3)	C8A—C9A	1.535 (3)
C8B—C14B	1.521 (3)	C8A—C14A	1.518 (3)
C9B—H9B	1.0000	C9A—H9A	1.0000
C9B—C10B	1.556 (3)	C9A—C10A	1.560 (3)
C9B—C11B	1.539 (3)	C9A—C11A	1.544 (3)
C10B—C19B	1.542 (3)	C10A—C19A	1.540 (3)
C11B—H11C	0.9900	C11A—H11A	0.9900
C11B—H11D	0.9900	C11A—H11B	0.9900
C11B—C12B	1.536 (3)	C11A—C12A	1.547 (3)
C12B—H12C	0.9900	C12A—H12A	0.9900
C12B—H12D	0.9900	C12A—H12B	0.9900
C12B—C13B	1.533 (3)	C12A—C13A	1.531 (3)
C13B—C14B	1.544 (3)	C13A—C14A	1.547 (3)
C13B—C17B	1.559 (3)	C13A—C17A	1.566 (3)
C13B—C18B	1.534 (3)	C13A—C18A	1.538 (3)
C14B—H14B	1.0000	C14A—H14A	1.0000
C14B—C15B	1.532 (3)	C14A—C15A	1.528 (3)
C15B—H15C	0.9900	C15A—H15A	0.9900
C15B—H15D	0.9900	C15A—H15B	0.9900
C15B—C16B	1.550 (3)	C15A—C16A	1.542 (3)
C16B—H16C	0.9900	C16A—H16A	0.9900
C16B—H16D	0.9900	C16A—H16B	0.9900
C16B—C17B	1.551 (3)	C16A—C17A	1.551 (3)
C17B—H17B	1.0000	C17A—H17A	1.0000
C17B—C20B	1.539 (3)	C17A—C20A	1.543 (3)
C18B—H18D	0.9800	C18A—H18A	0.9800
C18B—H18E	0.9800	C18A—H18B	0.9800
C18B—H18F	0.9800	C18A—H18C	0.9800
C19B—H19D	0.9800	C19A—H19A	0.9800
C19B—H19E	0.9800	C19A—H19B	0.9800
C19B—H19F	0.9800	C19A—H19C	0.9800
C20B—H20B	1.0000	C20A—H20A	1.0000
C20B—C21B	1.529 (3)	C20A—C21A	1.534 (3)
C20B—C22B	1.543 (3)	C20A—C22A	1.547 (3)
C21B—H21D	0.9800	C21A—H21A	0.9800
C21B—H21E	0.9800	C21A—H21B	0.9800
C21B—H21F	0.9800	C21A—H21C	0.9800
C22B—H22C	0.9900	C22A—H22A	0.9900
C22B—H22D	0.9900	C22A—H22B	0.9900
C22B—C23B	1.530 (3)	C22A—C23A	1.535 (3)
C23B—H23C	0.9900	C23A—H23A	0.9900
C23B—H23D	0.9900	C23A—H23B	0.9900
C23B—C24B	1.525 (3)	C23A—C24A	1.524 (3)
C24B—H24C	0.9900	C24A—H24A	0.9900
C24B—H24D	0.9900	C24A—H24B	0.9900
C24B—C25B	1.536 (3)	C24A—C25A	1.537 (3)
C25B—H25B	1.0000	C25A—H25A	1.0000
C25B—C26B	1.523 (4)	C25A—C26A	1.525 (4)
C25B—C27B	1.532 (3)	C25A—C27A	1.529 (4)
C26B—H26D	0.9800	C26A—H26A	0.9800
C26B—H26E	0.9800	C26A—H26B	0.9800
C26B—H26F	0.9800	C26A—H26C	0.9800
C27B—H27D	0.9800	C27A—H27A	0.9800
C27B—H27E	0.9800	C27A—H27B	0.9800
C27B—H27F	0.9800	C27A—H27C	0.9800
C28B—C29B	1.474 (3)	C28A—C29A	1.470 (3)
C28B—O1B	1.346 (3)	C28A—O1A	1.346 (3)
C28B—O2B	1.212 (3)	C28A—O2A	1.215 (3)
C29B—H29B	0.9500	C29A—H29A	0.9500
C29B—C30B	1.332 (3)	C29A—C30A	1.341 (3)
C30B—H30B	0.9500	C30A—H30A	0.9500
C30B—C31B	1.465 (3)	C30A—C31A	1.461 (3)
C31B—C32B	1.399 (3)	C31A—C32A	1.404 (3)
C31B—C36B	1.399 (3)	C31A—C36A	1.396 (3)
C32B—H32B	0.9500	C32A—H32A	0.9500
C32B—C33B	1.388 (3)	C32A—C33A	1.379 (3)
C33B—H33B	0.9500	C33A—H33A	0.9500
C33B—C34B	1.395 (3)	C33A—C34A	1.397 (3)
C34B—C35B	1.389 (3)	C34A—C35A	1.400 (3)
C34B—O3B	1.366 (3)	C34A—O3A	1.360 (3)
C35B—H35B	0.9500	C35A—H35A	0.9500
C35B—C36B	1.383 (3)	C35A—C36A	1.388 (3)
C36B—H36B	0.9500	C36A—H36A	0.9500
C37B—H37C	0.9900	C37A—H37A	0.9900
C37B—H37D	0.9900	C37A—H37B	0.9900
C37B—C38B	1.516 (3)	C37A—C38A	1.511 (3)
C37B—O3B	1.432 (3)	C37A—O3A	1.436 (3)
C38B—H38D	0.9800	C38A—H38A	0.9800
C38B—H38E	0.9800	C38A—H38B	0.9800
C38B—H38F	0.9800	C38A—H38C	0.9800
			
H1BA—C1B—H1BB	107.7	H1AA—C1A—H1AB	107.8
C2B—C1B—H1BA	108.8	C2A—C1A—H1AA	109.0
C2B—C1B—H1BB	108.8	C2A—C1A—H1AB	109.0
C2B—C1B—C10B	113.88 (17)	C2A—C1A—C10A	112.90 (19)
C10B—C1B—H1BA	108.8	C10A—C1A—H1AA	109.0
C10B—C1B—H1BB	108.8	C10A—C1A—H1AB	109.0
C1B—C2B—H2BA	109.8	C1A—C2A—H2AA	109.6
C1B—C2B—H2BB	109.8	C1A—C2A—H2AB	109.6
H2BA—C2B—H2BB	108.2	H2AA—C2A—H2AB	108.2
C3B—C2B—C1B	109.51 (18)	C3A—C2A—C1A	110.1 (2)
C3B—C2B—H2BA	109.8	C3A—C2A—H2AA	109.6
C3B—C2B—H2BB	109.8	C3A—C2A—H2AB	109.6
C2B—C3B—H3B	109.6	C2A—C3A—H3A	109.9
C2B—C3B—C4B	111.37 (18)	C4A—C3A—C2A	111.1 (2)
C4B—C3B—H3B	109.6	C4A—C3A—H3A	109.9
O1B—C3B—C2B	106.53 (17)	O1A—C3A—C2A	109.28 (18)
O1B—C3B—H3B	109.6	O1A—C3A—H3A	109.9
O1B—C3B—C4B	109.97 (17)	O1A—C3A—C4A	106.86 (18)
C3B—C4B—H4BA	109.4	C3A—C4A—H4AA	109.3
C3B—C4B—H4BB	109.4	C3A—C4A—H4AB	109.3
H4BA—C4B—H4BB	108.0	C3A—C4A—C5A	111.79 (19)
C5B—C4B—C3B	111.38 (17)	H4AA—C4A—H4AB	107.9
C5B—C4B—H4BA	109.4	C5A—C4A—H4AA	109.3
C5B—C4B—H4BB	109.4	C5A—C4A—H4AB	109.3
C4B—C5B—C10B	116.27 (18)	C4A—C5A—C10A	116.48 (17)
C6B—C5B—C4B	120.64 (19)	C6A—C5A—C4A	120.1 (2)
C6B—C5B—C10B	123.06 (18)	C6A—C5A—C10A	123.35 (19)
C5B—C6B—H6B	117.4	C5A—C6A—H6A	117.7
C5B—C6B—C7B	125.2 (2)	C5A—C6A—C7A	124.6 (2)
C7B—C6B—H6B	117.4	C7A—C6A—H6A	117.7
C6B—C7B—H7BA	109.2	C6A—C7A—H7AA	109.1
C6B—C7B—H7BB	109.2	C6A—C7A—H7AB	109.1
C6B—C7B—C8B	111.99 (17)	C6A—C7A—C8A	112.57 (19)
H7BA—C7B—H7BB	107.9	H7AA—C7A—H7AB	107.8
C8B—C7B—H7BA	109.2	C8A—C7A—H7AA	109.1
C8B—C7B—H7BB	109.2	C8A—C7A—H7AB	109.1
C7B—C8B—H8B	108.8	C7A—C8A—H8A	108.7
C7B—C8B—C9B	109.31 (17)	C7A—C8A—C9A	109.95 (19)
C9B—C8B—H8B	108.8	C9A—C8A—H8A	108.7
C14B—C8B—C7B	111.26 (17)	C14A—C8A—C7A	110.50 (18)
C14B—C8B—H8B	108.8	C14A—C8A—H8A	108.7
C14B—C8B—C9B	109.81 (16)	C14A—C8A—C9A	110.34 (18)
C8B—C9B—H9B	106.4	C8A—C9A—H9A	106.5
C8B—C9B—C10B	111.54 (16)	C8A—C9A—C10A	111.39 (18)
C10B—C9B—H9B	106.4	C8A—C9A—C11A	112.11 (17)
C11B—C9B—C8B	113.04 (17)	C10A—C9A—H9A	106.5
C11B—C9B—H9B	106.4	C11A—C9A—H9A	106.5
C11B—C9B—C10B	112.56 (17)	C11A—C9A—C10A	113.40 (17)
C1B—C10B—C9B	108.67 (16)	C1A—C10A—C9A	108.91 (18)
C5B—C10B—C1B	108.76 (17)	C5A—C10A—C1A	108.35 (18)
C5B—C10B—C9B	109.33 (17)	C5A—C10A—C9A	110.11 (16)
C5B—C10B—C19B	109.07 (17)	C5A—C10A—C19A	108.81 (19)
C19B—C10B—C1B	109.34 (18)	C19A—C10A—C1A	109.54 (19)
C19B—C10B—C9B	111.62 (17)	C19A—C10A—C9A	111.08 (18)
C9B—C11B—H11C	108.6	C9A—C11A—H11A	108.7
C9B—C11B—H11D	108.6	C9A—C11A—H11B	108.7
H11C—C11B—H11D	107.6	C9A—C11A—C12A	114.31 (17)
C12B—C11B—C9B	114.64 (18)	H11A—C11A—H11B	107.6
C12B—C11B—H11C	108.6	C12A—C11A—H11A	108.7
C12B—C11B—H11D	108.6	C12A—C11A—H11B	108.7
C11B—C12B—H12C	109.4	C11A—C12A—H12A	109.3
C11B—C12B—H12D	109.4	C11A—C12A—H12B	109.3
H12C—C12B—H12D	108.0	H12A—C12A—H12B	107.9
C13B—C12B—C11B	111.00 (17)	C13A—C12A—C11A	111.66 (18)
C13B—C12B—H12C	109.4	C13A—C12A—H12A	109.3
C13B—C12B—H12D	109.4	C13A—C12A—H12B	109.3
C12B—C13B—C14B	105.91 (16)	C12A—C13A—C14A	106.12 (17)
C12B—C13B—C17B	117.08 (17)	C12A—C13A—C17A	117.86 (18)
C12B—C13B—C18B	111.16 (18)	C12A—C13A—C18A	110.86 (18)
C14B—C13B—C17B	100.30 (16)	C14A—C13A—C17A	99.16 (16)
C18B—C13B—C14B	112.33 (17)	C18A—C13A—C14A	112.57 (18)
C18B—C13B—C17B	109.57 (17)	C18A—C13A—C17A	109.73 (18)
C8B—C14B—C13B	114.59 (17)	C8A—C14A—C13A	115.12 (17)
C8B—C14B—H14B	106.5	C8A—C14A—H14A	106.1
C8B—C14B—C15B	117.44 (16)	C8A—C14A—C15A	117.55 (19)
C13B—C14B—H14B	106.5	C13A—C14A—H14A	106.1
C15B—C14B—C13B	104.70 (16)	C15A—C14A—C13A	105.03 (17)
C15B—C14B—H14B	106.5	C15A—C14A—H14A	106.1
C14B—C15B—H15C	110.9	C14A—C15A—H15A	110.9
C14B—C15B—H15D	110.9	C14A—C15A—H15B	110.9
C14B—C15B—C16B	104.28 (16)	C14A—C15A—C16A	104.10 (18)
H15C—C15B—H15D	108.9	H15A—C15A—H15B	109.0
C16B—C15B—H15C	110.9	C16A—C15A—H15A	110.9
C16B—C15B—H15D	110.9	C16A—C15A—H15B	110.9
C15B—C16B—H16C	110.3	C15A—C16A—H16A	110.3
C15B—C16B—H16D	110.3	C15A—C16A—H16B	110.3
C15B—C16B—C17B	106.99 (16)	C15A—C16A—C17A	106.92 (17)
H16C—C16B—H16D	108.6	H16A—C16A—H16B	108.6
C17B—C16B—H16C	110.3	C17A—C16A—H16A	110.3
C17B—C16B—H16D	110.3	C17A—C16A—H16B	110.3
C13B—C17B—H17B	107.3	C13A—C17A—H17A	107.3
C16B—C17B—C13B	102.90 (16)	C16A—C17A—C13A	103.59 (17)
C16B—C17B—H17B	107.3	C16A—C17A—H17A	107.3
C20B—C17B—C13B	118.39 (17)	C20A—C17A—C13A	119.42 (17)
C20B—C17B—C16B	113.14 (17)	C20A—C17A—C16A	111.31 (18)
C20B—C17B—H17B	107.3	C20A—C17A—H17A	107.3
C13B—C18B—H18D	109.5	C13A—C18A—H18A	109.5
C13B—C18B—H18E	109.5	C13A—C18A—H18B	109.5
C13B—C18B—H18F	109.5	C13A—C18A—H18C	109.5
H18D—C18B—H18E	109.5	H18A—C18A—H18B	109.5
H18D—C18B—H18F	109.5	H18A—C18A—H18C	109.5
H18E—C18B—H18F	109.5	H18B—C18A—H18C	109.5
C10B—C19B—H19D	109.5	C10A—C19A—H19A	109.5
C10B—C19B—H19E	109.5	C10A—C19A—H19B	109.5
C10B—C19B—H19F	109.5	C10A—C19A—H19C	109.5
H19D—C19B—H19E	109.5	H19A—C19A—H19B	109.5
H19D—C19B—H19F	109.5	H19A—C19A—H19C	109.5
H19E—C19B—H19F	109.5	H19B—C19A—H19C	109.5
C17B—C20B—H20B	108.0	C17A—C20A—H20A	108.1
C17B—C20B—C22B	110.32 (17)	C17A—C20A—C22A	110.02 (18)
C21B—C20B—C17B	111.76 (18)	C21A—C20A—C17A	111.94 (19)
C21B—C20B—H20B	108.0	C21A—C20A—H20A	108.1
C21B—C20B—C22B	110.54 (19)	C21A—C20A—C22A	110.57 (19)
C22B—C20B—H20B	108.0	C22A—C20A—H20A	108.1
C20B—C21B—H21D	109.5	C20A—C21A—H21A	109.5
C20B—C21B—H21E	109.5	C20A—C21A—H21B	109.5
C20B—C21B—H21F	109.5	C20A—C21A—H21C	109.5
H21D—C21B—H21E	109.5	H21A—C21A—H21B	109.5
H21D—C21B—H21F	109.5	H21A—C21A—H21C	109.5
H21E—C21B—H21F	109.5	H21B—C21A—H21C	109.5
C20B—C22B—H22C	108.7	C20A—C22A—H22A	108.6
C20B—C22B—H22D	108.7	C20A—C22A—H22B	108.6
H22C—C22B—H22D	107.6	H22A—C22A—H22B	107.5
C23B—C22B—C20B	114.32 (19)	C23A—C22A—C20A	114.86 (19)
C23B—C22B—H22C	108.7	C23A—C22A—H22A	108.6
C23B—C22B—H22D	108.7	C23A—C22A—H22B	108.6
C22B—C23B—H23C	109.1	C22A—C23A—H23A	109.1
C22B—C23B—H23D	109.1	C22A—C23A—H23B	109.1
H23C—C23B—H23D	107.9	H23A—C23A—H23B	107.8
C24B—C23B—C22B	112.40 (19)	C24A—C23A—C22A	112.53 (19)
C24B—C23B—H23C	109.1	C24A—C23A—H23A	109.1
C24B—C23B—H23D	109.1	C24A—C23A—H23B	109.1
C23B—C24B—H24C	108.7	C23A—C24A—H24A	108.6
C23B—C24B—H24D	108.7	C23A—C24A—H24B	108.6
C23B—C24B—C25B	114.41 (19)	C23A—C24A—C25A	114.57 (19)
H24C—C24B—H24D	107.6	H24A—C24A—H24B	107.6
C25B—C24B—H24C	108.7	C25A—C24A—H24A	108.6
C25B—C24B—H24D	108.7	C25A—C24A—H24B	108.6
C24B—C25B—H25B	107.7	C24A—C25A—H25A	107.8
C26B—C25B—C24B	112.1 (2)	C26A—C25A—C24A	112.0 (2)
C26B—C25B—H25B	107.7	C26A—C25A—H25A	107.8
C26B—C25B—C27B	110.6 (2)	C26A—C25A—C27A	111.0 (2)
C27B—C25B—C24B	110.86 (19)	C27A—C25A—C24A	110.2 (2)
C27B—C25B—H25B	107.7	C27A—C25A—H25A	107.8
C25B—C26B—H26D	109.5	C25A—C26A—H26A	109.5
C25B—C26B—H26E	109.5	C25A—C26A—H26B	109.5
C25B—C26B—H26F	109.5	C25A—C26A—H26C	109.5
H26D—C26B—H26E	109.5	H26A—C26A—H26B	109.5
H26D—C26B—H26F	109.5	H26A—C26A—H26C	109.5
H26E—C26B—H26F	109.5	H26B—C26A—H26C	109.5
C25B—C27B—H27D	109.5	C25A—C27A—H27A	109.5
C25B—C27B—H27E	109.5	C25A—C27A—H27B	109.5
C25B—C27B—H27F	109.5	C25A—C27A—H27C	109.5
H27D—C27B—H27E	109.5	H27A—C27A—H27B	109.5
H27D—C27B—H27F	109.5	H27A—C27A—H27C	109.5
H27E—C27B—H27F	109.5	H27B—C27A—H27C	109.5
O1B—C28B—C29B	110.6 (2)	O1A—C28A—C29A	111.56 (19)
O2B—C28B—C29B	125.6 (2)	O2A—C28A—C29A	124.9 (2)
O2B—C28B—O1B	123.8 (2)	O2A—C28A—O1A	123.6 (2)
C28B—C29B—H29B	119.9	C28A—C29A—H29A	120.1
C30B—C29B—C28B	120.2 (2)	C30A—C29A—C28A	119.9 (2)
C30B—C29B—H29B	119.9	C30A—C29A—H29A	120.1
C29B—C30B—H30B	116.3	C29A—C30A—H30A	115.7
C29B—C30B—C31B	127.4 (2)	C29A—C30A—C31A	128.7 (2)
C31B—C30B—H30B	116.3	C31A—C30A—H30A	115.7
C32B—C31B—C30B	123.1 (2)	C32A—C31A—C30A	118.1 (2)
C32B—C31B—C36B	117.71 (19)	C36A—C31A—C30A	124.1 (2)
C36B—C31B—C30B	119.1 (2)	C36A—C31A—C32A	117.68 (19)
C31B—C32B—H32B	119.2	C31A—C32A—H32A	119.2
C33B—C32B—C31B	121.6 (2)	C33A—C32A—C31A	121.6 (2)
C33B—C32B—H32B	119.2	C33A—C32A—H32A	119.2
C32B—C33B—H33B	120.3	C32A—C33A—H33A	120.1
C32B—C33B—C34B	119.3 (2)	C32A—C33A—C34A	119.9 (2)
C34B—C33B—H33B	120.3	C34A—C33A—H33A	120.1
C35B—C34B—C33B	120.1 (2)	C33A—C34A—C35A	119.6 (2)
O3B—C34B—C33B	124.3 (2)	O3A—C34A—C33A	115.1 (2)
O3B—C34B—C35B	115.6 (2)	O3A—C34A—C35A	125.3 (2)
C34B—C35B—H35B	120.1	C34A—C35A—H35A	120.2
C36B—C35B—C34B	119.8 (2)	C36A—C35A—C34A	119.6 (2)
C36B—C35B—H35B	120.1	C36A—C35A—H35A	120.2
C31B—C36B—H36B	119.3	C31A—C36A—H36A	119.2
C35B—C36B—C31B	121.4 (2)	C35A—C36A—C31A	121.6 (2)
C35B—C36B—H36B	119.3	C35A—C36A—H36A	119.2
H37C—C37B—H37D	108.6	H37A—C37A—H37B	108.6
C38B—C37B—H37C	110.3	C38A—C37A—H37A	110.4
C38B—C37B—H37D	110.3	C38A—C37A—H37B	110.4
O3B—C37B—H37C	110.3	O3A—C37A—H37A	110.4
O3B—C37B—H37D	110.3	O3A—C37A—H37B	110.4
O3B—C37B—C38B	107.1 (2)	O3A—C37A—C38A	106.4 (2)
C37B—C38B—H38D	109.5	C37A—C38A—H38A	109.5
C37B—C38B—H38E	109.5	C37A—C38A—H38B	109.5
C37B—C38B—H38F	109.5	C37A—C38A—H38C	109.5
H38D—C38B—H38E	109.5	H38A—C38A—H38B	109.5
H38D—C38B—H38F	109.5	H38A—C38A—H38C	109.5
H38E—C38B—H38F	109.5	H38B—C38A—H38C	109.5
C28B—O1B—C3B	116.80 (18)	C28A—O1A—C3A	116.53 (18)
C34B—O3B—C37B	118.36 (19)	C34A—O3A—C37A	118.18 (18)

### Hydrogen-bond geometry (Å, º)

**Table:**

*D*—H···*A*	*D*—H	H···*A*	*D*···*A*	*D*—H···*A*
C33*B*—H33*B*···O2*B*^i^	0.95	2.56	3.389 (3)	145
C37*B*—H37*C*···O2*B*^i^	0.99	2.59	3.425 (3)	142
C37*A*—H37*B*···O2*A*^ii^	0.99	2.40	3.362 (3)	163

Symmetry codes: (i) −*x*+2, *y*+1/2, −*z*+2; (ii) −*x*+1, *y*−1/2, −*z*+1.

### Cremer and Pople puckering parameters (Q, θ, φ)

**Table:**

Ring	Atoms	Q(Å )i	θ (°)i	φ (°)i	Q(Å )ii	θ (°)ii	φ (°)ii
A	(C1-C2-C3-C4-C5-C10)i,ii	0.551 (3)	7.4 (2)	58 (2)	0.546 (2)	7.0 (2)	73.3 (19)
B	(C5-C6-C7-C8-C9-C10)i,ii	0.496 (2)	51.2 (3)	211.8 (4)	0.509 (2)	51.1 (2)	213.9 (3)
C	(C8-C9-C11-C12-C13-C14)i,ii	0.560 (2)	8.7 (2)	250.9 (16)	0.565 (2)	11.2 (2)	249.6 (11)
D	(C13-C14-C15-C16-C17)i,ii	0.457 (2)	-	186.7 (3)	0.452 (2)	-	184.1 (3)

i: molecule A; ii: molecule B.

## References

[bb1] Bruker (2009). *APEX2*, *SAINT* and *SADABS* Bruker AXS Inc., Madison, Wisconsin, USA.

[bb2] Cremer, D. & Pople, J. A. (1975). *J. Am. Chem. Soc.* **97**, 1354–1358.

[bb3] Dolomanov, O. V., Bourhis, L. J., Gildea, R. J., Howard, J. A. K. & Puschmann, H. (2009). *J. Appl. Cryst.* **42**, 339–341.

[bb4] Dong, X., Guo, J. & Wei, J. (2010). *Chin. J. Chem. Phys.* **23**, 719–725.

[bb5] Kutulya, L. A., Cherkashina, R. M., Tishchenko, V. G., Surov, Yu. N. & Polishchuk, A. G. (1983). *Zh. Obshch. Khim.* **53**, 1665–1668.

[bb6] Macrae, C. F., Bruno, I. J., Chisholm, J. A., Edgington, P. R., McCabe, P., Pidcock, E., Rodriguez-Monge, L., Taylor, R., van de Streek, J. & Wood, P. A. (2008). *J. Appl. Cryst.* **41**, 466–470.

[bb7] Sheldrick, G. M. (2008). *Acta Cryst.* A**64**, 112–122. 10.1107/S0108767307043930 18156677

[bb8] Siri, D., Siri, A. G. & Tordo, P. (2002). *J. Mol. Struct.* **582**, 171–185.

[bb9] Spek, A. L. (2009). *Acta Cryst.* D**65**, 148–155. 10.1107/S090744490804362X PMC2631630 19171970

[bb10] Tanaka, Y., Tsuchiya, H., Suzuki, M., Tsuda, K., Takano, J. & Kurihara, H. (1981). *Mol. Cryst. Liq. Cryst.* **68**, 113–125.

[bb11] Thiemann, T., al-Sulaibi, M., Al-Jasem, Y. & al-Hindawi, B. (2011). *Proc. 15th Int. Electron. Conf. Synth. Org. Chem.* 1–30 November 2011. Sciforum Electronic Conferences Series.

[bb12] Vora, R. A. (1976). *Curr. Sci.* **45**, 538–539.

